# Enhancing the Immunogenicity of Vaccinia Virus

**DOI:** 10.3390/v14071453

**Published:** 2022-06-30

**Authors:** Sergei N. Shchelkunov, Stanislav N. Yakubitskiy, Alexander A. Sergeev, Ekaterina V. Starostina, Ksenia A. Titova, Stepan A. Pyankov, Galina A. Shchelkunova, Mariya B. Borgoyakova, Alexey M. Zadorozhny, Lyubov A. Orlova, Denis N. Kisakov, Larisa I. Karpenko

**Affiliations:** State Research Center of Virology and Biotechnology VECTOR, Rospotrebnadzor, 630559 Koltsovo, Novosibirsk Region, Russia; yakubitskiy_sn@vector.nsc.ru (S.N.Y.); sergeev_ala@vector.nsc.ru (A.A.S.); starostina_ev@vector.nsc.ru (E.V.S.); titova_ka@vector.nsc.ru (K.A.T.); pyankov_sa@vector.nsc.ru (S.A.P.); galina_shchelkunova@rambler.ru (G.A.S.); borgoyakova_mb@vector.nsc.ru (M.B.B.); lexazzador@mail.ru (A.M.Z.); orlova_la@vector.nsc.ru (L.A.O.); def_2003@mail.ru (D.N.K.); lkarpenko@ngs.ru (L.I.K.)

**Keywords:** poxviruses, vaccinia virus, vaccines, adaptive immune response, antibodies, T cells

## Abstract

The conventional live smallpox vaccine based on the vaccinia virus (VACV) cannot be widely used today because it is highly reactogenic. Therefore, there is a demand for designing VACV variants possessing enhanced immunogenicity, making it possible to reduce the vaccine dose and, therefore, significantly eliminate the pathogenic effect of the VACV on the body. In this study, we analyzed the development of the humoral and T cell-mediated immune responses elicited by immunizing mice with low-dose VACV variants carrying the mutant *A34R* gene (which increases production of extracellular virions) or the deleted *A35R* gene (whose protein product inhibits antigen presentation by the major histocompatibility complex class II). The VACV LIVP strain, which is used as a smallpox vaccine in Russia, and its recombinant variants LIVP-A34R*, LIVP-dA35R, and LIVP-A34R*-dA35R, were compared upon intradermal immunization of BALB/c mice at a dose of 10^4^ pfu/animal. The strongest T cell-mediated immunity was detected in mice infected with the LIVP-A34R*-dA35R virus. The parental LIVP strain induced a significantly lower antibody level compared to the strains carrying the modified *A34R* and *A35R* genes. Simultaneous modification of the *A34R* gene and deletion of the *A35R* gene in VACV LIVP synergistically enhanced the immunogenic properties of the LIVP-A34R*-dA35R virus.

## 1. Introduction

The largest and most complexly organized mammalian DNA viruses are combined into the subfamily Chordopoxvirinae, in the family Poxviridae. The best-studied species are those belonging to the genus *Orthopoxvirus*; the reason is that this genus includes human pathogenic species such as *Variola virus* (VARV), *Monkeypox virus* (MPXV), *Cowpox virus* (CPXV), and *Vaccinia virus* (VACV). Different species belonging to the genus *Orthopoxvirus* are indiscernible in terms of virion morphology, are related to each other both antigenically and immunologically, exhibit serological cross-reactivity, and elicit cross-reactive immunity [1].

Smallpox epidemics led to the death of up to 30–40% of people infected [2]. The very first immunoprophylaxis methods have been elaborated to ensure protection against smallpox. The first method used was the intradermal (transepidermal) inoculation of a preparation containing the causative agent of smallpox, the variola virus (VARV). This procedure became known as *variola inoculation* or *variolation*. However, this protection method ended up with the death of patients relatively often (0.5–2% of the total number of immunized patients); therefore, it did not become commonplace. The situation changed drastically after, in 1796, E. Jenner introduced into practice the inoculation of infectious material taken from cows or horses with smallpox-like disease, as well as humans to whom this infection had been transmitted from these domestic animals, for the prevention of smallpox infection. This procedure became known as *vaccine inoculation*, and later, as *vaccination* [3]. This vaccination had an extremely rare mortality rate and ensured sufficiently reliable protection against smallpox. It was believed for a long time that CPXV preparations were used as a smallpox vaccine. However, in the 20th century, it was proved that the less reactogenic VACV, closely related to horsepox virus, was actually used [4].

Along with the inestimable benefit of mass smallpox vaccination for humanity, its application was accompanied by post-vaccination complications. Neurological complications (10–300 cases per 1,000,000 of vaccinated people, depending on age) were the most severe ones; ~10% of those resulted in death [2,5,6]. Therefore, after the global eradication of smallpox had been certified, in 1980, the World Health Organization (WHO) strongly recommended that smallpox vaccination was stopped in all countries [2].

Because of the cessation of mass smallpox vaccination, over the past decades, humans have lost the herd immunity against both smallpox and other orthopoxvirus infections. Therefore, the risk of global transmission of zoonotic orthopoxvirus infections primarily caused by MPXV or CPXV to humans has increased [4,7].

First-generation VACV-based smallpox vaccines are reactogenic. Therefore, these vaccines cannot be used today for mass immunization as the percentage of immunocompromised people (HIV-infected individuals, cancer patients, etc.) has substantially increased. Therefore, there is a demand for designing modern, safe, VACV-based live vaccines using genetic engineering methods [8]. In particular, designing VACV variants with enhanced immunogenicity can reduce the virus dose needed for vaccination, thus significantly decreasing its potential pathogenic effect on the body [3,9].

This study examines the development of humoral and cell-mediated immunities elicited in mice after an intradermal injection of low-dose VACV variants carrying the target mutated *A34R* gene (which enhances the production of extracellular virions) or the deleted *A35R* gene (which controls antigen presentation by the major histocompatibility complex class II), as well as the VACV carrying a combination of these two mutations.

## 2. Materials and Methods

### 2.1. Viruses and Cells

The clonal variant 14 of the LIVP VACV strain [10], as well as the LIVP-A34R* [11] and LIVP-dA35R strains [12] based on it, were used in this study. The CPXV strain GRI-90 [13] was obtained from a virus collection; the continuous CV-1 African green monkey kidney cell line was obtained from the cell culture collection of the State Research Center of Virology and Biotechnology (SRC VB) VECTOR. The viruses were grown and titrated on the CV-1 cell culture according to the procedure described in [14].

### 2.2. Animals

Female BALB/c mice (weight, 16–19 g) from the breeding nursery of SRC VB VECTOR were used in the studies. The experimental animals were fed the standard diet, with sufficient amount of water in compliance with the veterinary regulations and the requirements for humane handling and use of animals in experimental research. Animal manipulations were approved by the Bioethics Committee of SRC VB VECTOR (Protocol No. 01-04.2021, dated 22 April 2021).

### 2.3. Infecting Mice

The preparations of LIVP, LIVP-A34R*, LIVP-dA35R, and LIVP-A34R*-dA35R viruses (dose 10^4^ pfu/animal) were inoculated intradermally (i.d.) to mice by injecting the infectious material or saline solution (0.02 mL) into the tail. Each group consisted of 12 mice.

The injection site (the dorsal side of the tail, ~1 cm from the tail base area) was pre-treated with 70% ethanol; a 30G needle (0.3 mm × 13 mm) was inserted at a small angle (bevel-up position) to a depth of ~2–3 mm under the outermost epidermis layer. The viral material/saline solution was injected slowly as we anticipated that the epidermis would become stratified due to pressure exerted by the injected fluid (skin blanching spreading bilaterally from the injection site indicated that fluid had got into the intradermal space). The needle was retracted slowly after the injection; the injection site was disinfected with 70% ethanol.

### 2.4. Sampling of Biomaterial from the Experimental Animals

Blood samples were collected from the retro-orbital venous sinus by puncturing it with a 23G needle (0.6 mm × 30 mm) 14 days after the injection of VACV preparation in mice (six animals per group); the animals were then euthanized by cervical dislocation. Spleens for splenocyte isolation were removed under sterile conditions using tweezers and surgical scissors, and were then placed into the transport medium.

Serum samples were obtained from individual blood samples of mice by precipitation of blood corpuscles via centrifugation. Mouse serum samples were stored at −20 °C.

On day 28 after the VACV preparations had been injected, blood samples were collected intravitally from the retro-orbital venous sinus of mice (six animals per group), and individual serum samples were obtained using the procedure described above.

### 2.5. Enzyme-Linked Immunosorbent Assay of Serum Samples

ELISA of individual mouse serum samples was performed according to the procedure described in [14]. Purified VACV LIVP preparation was used as an antigen. The geometric means of logs of the reciprocal titer of VACV-specific IgM and IgG across the study groups were determined, and the 95% confidence intervals for the probability of matching between each sample and the parent population were calculated.

### 2.6. Splenocyte Isolation

Spleens collected from immunized mice were mashed through 70 μm and 40 μm cell strainers (BD Falcon™, Tewksbury, MA, USA). Splenocytes were treated with red blood cell lysis buffer (ACK Lysis Buffer, Sigma, St. Louis, MO, USA); then, cells were washed with completed RPMI 1640 medium and suspended in completed RPMI 1640 medium with 10% fetal bovine serum, 2 mM L-Gln, and 50 µg/mL gentamycin. Cells were counted using a TC20™ automated cell counter (Bio-Rad, Hercules, CA, USA).

### 2.7. IFN-γ ELISpot Assay

The assays were performed using a mouse IFN-γ ELISpot Kit (R&D Systems, Inc., Minneapolis, MN, USA) according to the manufacturer’s instructions. Splenocytes were plated (100 µL/well) in duplicate (5 × 10^6^ cells/mL) and stimulated by a mixture of peptides (corresponding to VACV-specific BALB/c mice H2-d restricted epitopes): SPYAAGYDL, SPGAAGYDL, VGPSNSPTF, KYGRLFNEI, GFIRSLQTI, and KYMWCYSQV [15,16]. Pooled peptides (100 µL/well) were added at a concentration of 20 µg/mL for each peptide. Non-stimulated and concanavalin A (Con A, 5 μg/mL)-stimulated splenocytes were used as negative and non-specific positive controls, respectively. After an 18-h stimulation period at 37 °C in 5% CO_2_, cells were discarded, and plates were incubated for 2 h at 37 °C with anti-IFN-γ detection antibodies.

Plates were washed and the spots were revealed by adding the streptavidin-conjugated alkaline phosphatase and BCIP/NBT (5-bromo-4-chloro-3′-indolylphosphate/nitro-blue tetrazolium) substrate. The reaction was stopped by washing plates with distilled water. The number of IFN-γ-secreting cells was counted using an ELISpot reader (Carl Zeiss, Jena, Germany).

### 2.8. Intracellular Cytokine Staining (ICS) Assays

ICS assay was performed according to a standard protocol, as described previously [17]. Briefly, splenocytes isolated from mice (2 × 10^6^ cells/well) were plated in 24-well plates and stimulated with a mix of VACV-specific peptides (SPYAAGYDL, SPGAAGYDL, VGPSNSPTF, KYGRLFNEI, GFIRSLQTI, and KYMWCYSQV) or with PMA (phorbol myristate acetate, 30 ng/mL) and ionomycin (1 µg/mL). Each peptide was added at a concentration of 20 µg/mL per well, and cells were incubated for 4 h at 37 °C in 5% CO_2_, and for additional 16 h with Brefeldin A (5 μg/mL, BD Biosciences, Franklin Lakes, NJ, USA). On the next day, cells were stained with pre-titrated anti-CD3 MCA500SBB700 (Bio-Rad, Hercules, CA, USA), anti-CD8 FITC (BD Pharmingen, San Diego, CA, USA), and anti-CD4 PerCP (BD Pharmingen, San Diego, CA, USA), fixed, and permeabilized using Cytofix/Cytoperm solution (BD Biosciences, Franklin Lakes, NJ, USA), according to the manufacturer’s instructions. Cells were then stained for intracellular cytokine detection with anti-IFN-γ APC (BD Pharmingen, San Diego, CA, USA). Samples were analyzed on a ZE5 flow cytometer (Bio-Rad, Hercules, CA, USA). Data were presented as the medians and ranges of variation.

### 2.9. Statistics

Data were analyzed using the GraphPad Prism 9.0 software (GraphPad Software, Inc., San Diego, CA, USA). Results are expressed as medians with range. Data were analyzed using non-parametric tests. Intergroup differences in immune responses were assessed with the non-parametric Mann–Whitney U test and one-way Kruskal–Wallis analysis of variance, adjusted for multiple comparisons used Dunn’s statistical hypothesis testing. The statistical analysis was conducted at a 95% confidence level. A *p* value less than 0.05 was considered statistically significant.

## 3. Results

### 3.1. Constructing VACV Carrying Single-Point Mutations in the A34R Genes and Deletion of the A35R Gene

**Figure 1:**
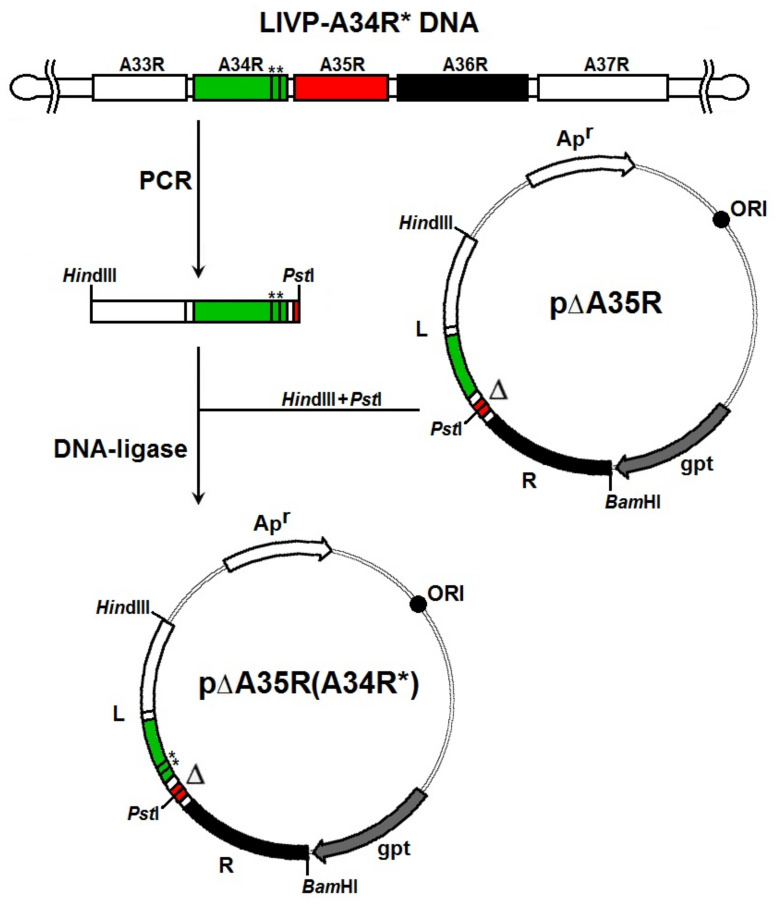
Scheme of construction of the plasmid p∆A35R(A34R*) (see explanation in the text). Vertical lines and asterisks indicate point mutations in *A34R* gene. L and R—left and right flanking *A35R* gene regions (**, *Vertical lines and asterisks indicate point mutations in A34R gene*).

To construct the VACV LIVP variant simultaneously carrying target mutations in the adjacent *A34R* and *A35R* genes, we produced the plasmid p∆A35R(A34R*) to be integrated into the viral genome according to the scheme shown in Figure 1.

For PCR amplification of the target fragment of the *A35R* left-flanking region, we used viral DNA of the earlier produced mutant LIVP-A34R* [11] (two substitutions in the *A34R* gene: D110N (nucleotide 328 in the gene, G→A) and K151E (nucleotide 451 in the gene, A→G)), isolated using the DNA-sorb-B kit (Central Research Institute of Epidemiology, Moscow, Russia); the following primers were used:

[5′-GGGG**AAGCTT**TCATGATGACACCAGAAAACGACGAA-3′ (***Hin*dIII**) and 5′-GCAA**CTGCAG**ATGGCGGCGTACGTTAACGACTTATT-3′ (***Pst*I**)] and Platinum *Taq* DNA High Fidelity Polymerase (Invitrogen, Waltham, MA, USA). The PCR product was purified using the PCR Purification Kit (QIAGEN, Hilden, Germany) and hydrolyzed with *Hin*dIII and *Pst*I restriction endonucleases (SibEnzyme, Novosibirsk, Russia); the resulting fragment was isolated from the reaction mixture by agarose gel electrophoresis using the QIAquick Gel Extraction Kit (QIAGEN, Hilden, Germany). The target fragment of the left-flanking sequence of the *A35R* gene was then incorporated into the purified *Hin*dIII-*Pst*I vector fragment of the plasmid p∆A35R created earlier [12] (Figure 1). Ligase mix was used for transforming chemically competent *E. coli* XL2-Blue cells; the transformant clones were selected on agar medium supplemented with ampicillin. The resulting plasmid was confirmed to have the correct sequence, and was designated p∆A35R(A34R*).

The recombinant strain LIVP-A34R*-dA35R was constructed by transfecting the VACV LIVP-A34R*-infected CV-1 cell culture with the plasmid p∆A35R(A34R*), using the procedure described earlier [10]. The correctness of mutations introduced to the *A34R* gene and deletion in the *A35R* gene in the selected LIVP-A34R*-dA35R variant was confirmed by sequencing.

### 3.2. Development of the Humoral Immune Response in Mice to Intradermal Infection with VACV Variants

**Figure 2:**
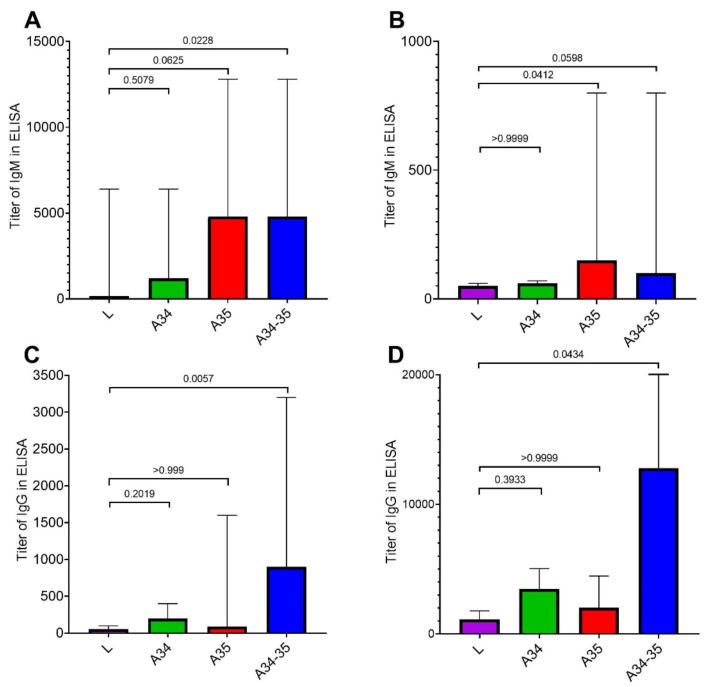
The VACV-specific humoral response in mice immunized with VACVs: L—the LIVP strain; A34—LIVP-A34R*; A35—LIVP-dA35R; and A34-35—LIVP-A34R*-dA35R. The reciprocal titers of virus-specific IgM on day 14 (**A**) and day 28 (**B**); the reciprocal titers of virus-specific IgG on day 14 (**C**) and day 28 (**D**) are given. The diagrams show the median values and the range. Statistical analysis was performed using the GraphPad Prism 9.0 software. Intergroup differences in immune responses were assessed with the non-parametric one-way Kruskal–Wallis analysis of variance adjusted for multiple comparisons used Dunn’s statistical hypothesis testing. The *p* values are indicated above the horizontal brackets.

The levels of VACV-specific IgM and IgG were assessed on days 14 and 28 after the intradermal (i.d.) inoculation of BALB/c mice with LIVP, LIVP-A34R*, LIVP-dA35R, or LIVP-A34R*-dA35R viruses at a dose of 10^4^ pfu/animal. The experimental findings (Figure 2) show that all the mutant variants induced a significantly higher level of IgM synthesis compared to the parental LIVP strain on day 14. The highest IgM level was revealed for the LIVP-dA35R and LIVP-A34R*-dA35R strains (Figure 2A).

Synthesis of VACV-specific IgG was also initiated by day 14 post-infection, although to a smaller extent compared to IgM production. The strongest IgG response was observed for the LIVP-A34R*-dA35R variant (Figure 2C).

On day 28, IgM synthesis declined significantly for all the analyzed VACV LIVP variants, still remaining the highest for the LIVP-dA35R and LIVP-A34R*-dA35R strains (Figure 2B). Meanwhile, the IgG level increased in all the groups of immunized mice: the highest IgG level was reached in animals immunized with LIVP-A34R*-dA35R (Figure 2D).

### 3.3. Analysis of the T Cell-Mediated Immune Response in Mice Infected with VACV Variants

Splenocytes were isolated from spleens of individual experimental animals on day 14 after they had been infected with parental LIVP or the mutant LIVP-A34R*, LIVP-dA35R, and LIVP-A34R*-dA35R variants. This sampling date was chosen because we had previously demonstrated that the maximum peak of T cell-mediated immune response is reached on day 14 after the i.d. infection of BALB/c mice with the VACV LIVP [17].

Intact (non-immunized) animals were used as controls. Samples taken from six animals were analyzed in each group. The T cell-mediated immune response was assessed by ELISpot assay and intracellular cytokine staining (ICS).

**Figure 3:**
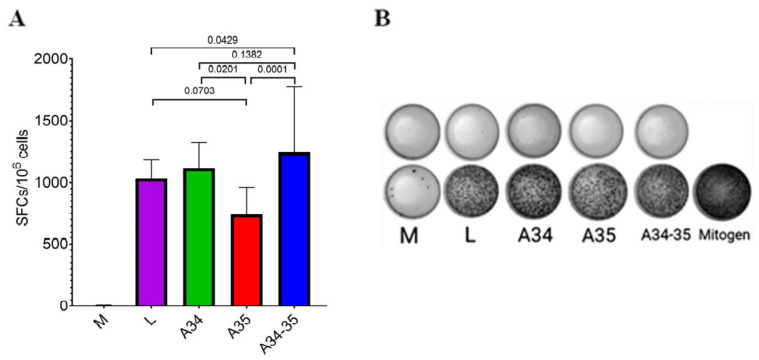
The results of the ELISpot assay of the VACV-specific T cell-mediated response in immunized BALB/c mice. M—control (non-immunized) mice; L—the VACV LIVP strain; A34—LIVP-A34R*; A35—LIVP-dA35R; and A34-35—LIVP-A34R*-dA35R. (**A**) The number of cells expressing IFN-γ in response to stimulation with a pool of VACV-specific peptides, per 1 × 10^6^ splenocytes. The diagrams show the median values and the range. Statistical analysis was performed using the GraphPad Prism 9.0 software. Intergroup differences in immune responses were assessed with the non-parametric one-way Kruskal–Wallis analysis of variance adjusted for multiple comparisons used Dunn’s statistical hypothesis testing. The *p* values are indicated above the horizontal brackets. (**B**) The representative images of ELISpot wells (the top row: splenocytes not stimulated by peptides; the bottom row: splenocytes stimulated with peptide pool or a mitogen).

The ELISpot assay has demonstrated that the largest number of IFN-γ-secreting cells in response to stimulation by virus-specific peptides was detected in mice infected with the LIVP-A34R*-dA35R virus (Figure 3). Among all experimental groups, the smallest number of IFN-γ-secreting cells was detected in the group of mice infected with the LIVP-dA35R mutant. Overall, the results of the ELISpot assay demonstrate that a strong post-vaccination T-cell immune response has developed in all four groups of animals immunized with both the parental LIVP strain and the mutant LIVP variants.

**Figure 4:**
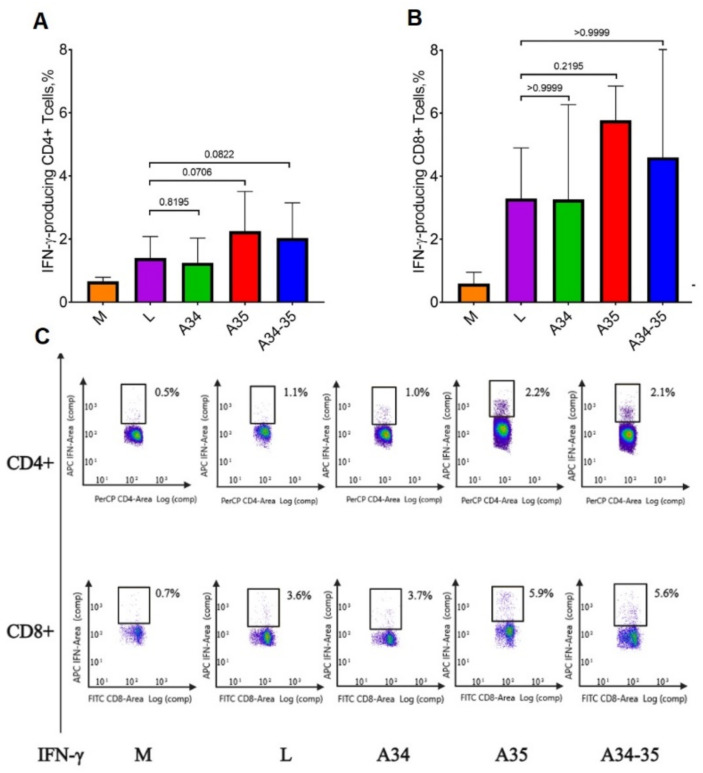
The results of analyzing the T cell-mediated response in immunized BALB/c mice using ICS and flow cytometry. M—control (non-immunized) mice; L—the VACV LIVP strain; A34—LIVP-A34R*; A35—LIVP-dA35R; and A34-35—LIVP-A34R*-dA35R. The percentages of virus-specific CD4+ (**A**) or CD8+ (**B**) T cells secreting IFN-γ in response to stimulation with a peptide pool from LIVP virus proteins are shown. The diagrams show the median values and the range. Statistical analysis was performed using the GraphPad Prism 9.0 software. Intergroup differences in immune responses were assessed with the non-parametric one-way Kruskal–Wallis analysis of variance adjusted for multiple comparisons used Dunn’s statistical hypothesis testing. The *p* values are indicated above the horizontal brackets. (**C**) The typical flow cytometry data plots (diagrams) for CD4+ and CD8+ T cells.

The assessments of T cell-mediated immune response by ICS (Figure 4) show that both helper T cells (CD4+) and cytotoxic T cells (CD8+) in animals immunized with all the LIVP variants were able to produce IFN-γ on specific viral peptide stimulation. Overall, the response of CD3+/CD8+ T cells is stronger compared to that of CD3+/CD4+ cells, which indicates the development of a pronounced virus-specific cytotoxic T-cell response.

Having compared the T cell-mediated responses to injection of different mutant LIVP variants, we can fairly say that in groups of animals immunized with LIVP-dA35R and LIVP-A34R*-dA35R, CD4+ cells were characterized by the highest level of IFN-γ secretion in response to specific stimulation (Figure 4A). This finding correlates positively with the ELISA data, which show an elevated level of specific IgM in these groups of mice (Figure 2A,B) and IgG in group of animals immunized with LIVP-A34R*-dA35R (Figure 2C,D).

Production of IFN-γ-secreting CD8+ T cells in the groups of mice infected with LIVP, LIVP-A34R*, LIVP-dA35R and LIVP-A34R*-dA35R had no statistically significant differences (Figure 4B).

## 4. Discussion

Mass smallpox vaccination and strict epidemiological surveillance have played a crucial role in the success achieved by the global smallpox eradication program conducted under the auspices of the WHO. This program was initiated in 1958; the last natural case of smallpox was recorded in October 1977. After the meticulous certification of smallpox eradication, the Thirty-Third World Health Assembly (WHA), held on 8 May 1980, solemnly adopted the declaration of global eradication of smallpox. For the first time in human history, due to the joint efforts of healthcare workers and scientists from many countries working under the WHO program, people attained freedom from the deadly infectious disease caused by the virus, which had been annually claiming millions of lives [1,2].

Different VACV strains were used as the live smallpox vaccine in various countries; the exact origin of these strains was unknown in most cases. These VACV strains differed in terms of their pathogenicity exhibited upon infection of different laboratory animal species, as well as in terms of reactogenicity during immunization of humans [1,6,18,19]. During the mass immunization campaign, all VACV strains caused severe adverse reactions in a small percentage of cases, including encephalitis and encephalomyelitis, which sometimes were lethal for the vaccinated people. Therefore, as early on as 14 May 1980, the Thirty-Third WHA adopted the resolution strongly encouraging all countries to stop smallpox vaccination of their residents [20].

As a result of cessation of smallpox vaccination, the vast majority of the world population currently has no immunity against smallpox or any other zoonotic orthopoxvirus infection. This creates a new situation involving the potential circulation of zoonotic orthopoxviruses in the human population, which may, therefore, alter the ecology and the range of susceptible hosts for different orthopoxvirus species [4]. For this reason, the outbreaks of diseases caused by zoonotic orthopoxviruses such as MPXV, CPXV, and VACV in humans have been reported increasingly often across different continents over the past years [21,22,23].

Methods of immunoprophylaxis need to be developed for preventing the transition of these outbreaks to large-scale epidemics, with preventive vaccination being the key one. Meanwhile, mass immunization with the conventional VACV-based vaccine is currently contraindicated because of its high reactogenicity. Therefore, there is a demand for designing VACV variants characterized by reduced virulence (attenuated) and/or enhanced immunogenicity/protective efficacy [19,24]. In the latter case, the immunizing dose of the virus can be significantly reduced, thus avoiding the pathogenic impact of VACV on the body [14].

VACV belongs to the genus *Orthopoxvirus*, family Poxviridae, which combines the largest mammalian DNA viruses. Depending on the orthopoxvirus species, its viral genome is 190–220 kbp long and encodes approximately 200 proteins. The intracellular mature virion (IMV) containing no less than 85 different viral proteins is the main infectious form in the progeny of these viruses. A small portion of the viral particles synthesized in the cell is coated into an additional lipoprotein envelope, and these extracellular enveloped virions (EEVs) exit the infected cells. EEVs additionally contain eight viral proteins associated with their outer membrane [3].

VACV-based vaccine preparations mainly contain IMV particles. Only in vivo replication of the VACV leads to production of anti-IMV and anti-EVV antibodies. Furthermore, only the live VACV in the animal or human body induces the synthesis of protective antibodies against non-virion proteins and stimulates the development of full-fledged cell-mediated immunity [24].

Because of the complex organization of orthopoxviruses, the mechanism of immune defense against smallpox (and other orthopoxvirus infections) has not yet been fully studied [3,9,24]. It has been proved that the humoral (antibody) immune response to a smallpox vaccine plays a crucial role in the defense against the subsequent orthopoxviral infection [14,15,16].

In most studies, the development of an adaptive immune response has been studied in a model of mice infected with the neurovirulent Western Reserve (WR) laboratory strain of VACV that has never been used for smallpox vaccination [15,25,26,27,28]. Therefore, the development of humoral and T-cell immunity in response to infection/vaccination with the virus should be studied for each other VACV vaccine strain.

The T cell immune response is mainly related to early proteins synthesized during VACV infection, while antibodies are largely synthesized in response to late viral proteins, both virion and non-virion ones. It is important to note that the T cell immune response, on the one hand, and the antibody response, on the other hand, respond to different VACV antigens and cover a wide range of viral proteins [3].

During their coevolution with susceptible animals, orthopoxviruses have developed various molecular mechanisms for suppressing different stages of development of the innate and adaptive immune responses to the infection. Deletion or targeted editing of viral genes suppressing the body’s immune response to infection may increase the immunogenicity of VACV in some cases [9,11].

The protein encoded by the VACV *A35R* gene was previously shown to inhibit antigen presentation by the major histocompatibility complex class II, thus reducing the antibody response to viral infection [12,29]. Importantly, among all the studied orthopoxvirus species, the ortholog of the VACV *A35R* gene is fragmented only in VARV [12]. This is probably one of the reasons why only infecting humans with VARV or variolation (immunization with the live VARV) ensures a life-long immunity against smallpox [2], while booster vaccination with VACV is needed to achieve a long-lasting immunity against this disease.

The *A34R* gene directs synthesis of a protein that is a component of the lipoprotein envelope of EEVs and controls the exit of this form of virions from cells and their efficient invasion of the infected organism. Most VACV strains produce less than 1% EEVs among the viral progeny when being replicated in cell cultures; however, the introduction of two single-point mutations to the *A34R* gene (nucleotide 328 in the coding region (G→A) and nucleotide 451 (A→G)) increases the proportion of EEVs in the viral progeny, thus enhancing the immune response to infection [11,30].

The pathogenicity and immunogenicity of VACV depend on the viral strain being used, as well as its route of administration and dose [14,31]. In most studies, the immunogenic properties of VACV variants were investigated by injecting mice with virus doses ranging from 10^6^ to 10^8^ pfu [11,14,25,30]. A decrease in the immunizing dose of VACV leads not only to a decrease in the reactogenicity of the virus, but also to a decrease in the level of synthesized VACV-specific antibodies [14,31].

In this work, we used a mouse model to study the effect of the *A34R* and *A35R* genes of the virus on the immunogenic properties of VACV LIVP at a low immunizing dose. It should be noted that VACV LIVP has been used in Russia as the first-generation smallpox vaccine.

The clonal variant of the LIVP strain and mutant strains based on it (LIVP-A34R* with two single-point target mutations introduced into the *A34R* gene, increasing EEV production [11]; LIVP-dA35R with the *A35R* gene deleted [12]; and the LIVP-A34R*-dA35R virus carrying a combination of both mutations) were compared. The viruses under study were injected intradermally (i.d.) to BALB/c mice at a dose of 10^4^ pfu.

Earlier, we revealed that the peak of the T cell-mediated immune response to infecting BALB/c mice with the LIVP virus is observed on day 14, while virus-specific IgG only start to be produced at this time; the IgG production reaches its maximum by day 28 [17]. Therefore, in this study, the level of T cell-mediated response of mice infected with VACV LIVP or its derivatives was assessed 14 days after the experiment had been started; the levels of IgM and IgG antibodies in these animals were analyzed on days 14 and 28.

In order to assess the induced protectivity in mice infected with the viruses under study, animals of experimental and control groups were infected intranasally (i.n.) with CPXV GRI-90 at a dose of 68 LD_50_ on day 30 (day 2 after blood samples had been collected). The animals were followed up for 14 days to document the events of their death. All the control (non-immunized) animals died on days 6–10, while neither clinical manifestation of CPXV infection nor death of animals were observed in all the groups of mice infected with VACV LIVP and the mutant variants being compared. We have demonstrated in another series of experiments that when BALB/c mice had been i.d. infected with the LIVP virus at dose 10^3^ pfu/animal, the virus ensured protective immunity in only 83% of animals i.n. infected with CPXV at a dose of 32 LD_50_, while all the mice immunized with LIVP-A34R*, LIVP-dA35R or LIVP-A34R*-dA35R remained alive [32]. These findings confirm that enhanced EEV production and/or prevention of viral A35 protein inhibition of antigen presentation by MHC class II glycoproteins increase the protectivity/immunogenicity of VACV.

The ELISpot assay data (Figure 3) confirmed the development of a strong post-vaccination T cell-mediated response in all four experimental groups of animals immunized with the LIVP, LIVP-A34R*, LIVP-dA35R, and LIVP-A34R*-dA35R strains. The strongest statistically significant T cell-mediated immune response was revealed only in mice infected with the LIVP-A34R*-dA35R virus.

An ICS assay demonstrated that immunization of mice with all four VACV variants elicited a stronger response of IFN-γ+ CD3+/CD8+ T cells compared to the response of IFN-γ+ CD3+/CD4+ T cells (Figure 4A,B), thus indicating that a pronounced virus-specific cytotoxic immune response had been developed in all cases. Statistically significant differences between the groups of mice in the level of production of both CD4+ and CD8+ VACV-specific IFN-γ-producing T cells could not be identified. It should be noted that the highest levels of IFN-γ-producing CD4+ T cells were observed in the groups of mice infected with LIVP-dA35R and LIVP-A34R*-dA35R strains (Figure 4A), but with low reliability.

ELISA of individual mouse serum samples showed (Figure 2) that the parental LIVP strain induced a statistically significantly lower level of virion-specific IgM and IgG antibodies compared to that of the virus strains simultaneously carrying the modified *A34R* and *A35R* genes. Strain LIVP-dA35R also induced high level IgM production (Figure 2A,B). In the case of animal immunization with LIVP-A34R* or LIVP-dA35R, the level of IgG immune response increased only slightly compared to immunization with the parental LIVP strain (Figure 2C,D).

Previously, a slight dose-dependent increase in the production of VACV-specific antibodies was observed with i.d. administration of the strain LIVP-A34R* to mice at doses of 10^6^–10^8^ pfu [11].

Our results are somewhat different from the published data for the neurovirulent VACV strain WR with individually deleted gene *A35R*. During i.n. infection of mice with strain WR with the deleted *A35R* gene, it was found that this mutant virus was attenuated [33], and elicited improved antibody and gamma interferon-secreting cell responses compared to the wild-type virus [34]. Unlike the WR strain, the strain LIVP studied in the present work does not lead to the death of mice when i.n. administered, even at a dose of 10^8^ pfu/animal [11]. It is possible that the difference between strains WR and LIVP in pathogenicity for mice, as well as the difference in routes of administration of viruses, lead to different results in the induction of an adaptive immune response of variants of these viruses with deletion of the *A35R* gene.

The summation of the results of the analysis of humoral and cellular immune response, developing after i.d. injection of the studied VACVs at a dose of 10^4^ pfu/mouse, give grounds for concluding that simultaneous modification of the *A34R* gene and deletion of the *A35R* gene in VACV LIVP synergistically enhanced the immunogenic properties of the LIVP-A34R*-dA35R virus. It can be assumed that this virus can be used for effective vaccination against orthopoxvirus infections (human monkeypox, cowpox, etc.) when injected intradermally at low doses, which will significantly reduce the reactogenicity of the vaccine strain.

## Data Availability

All raw data are available and provided upon request.
